# Feasibility of tension-free repair of inguinal hernia in senile patients under ultrasound-guided local nerve block

**DOI:** 10.1007/s13304-023-01747-6

**Published:** 2024-03-19

**Authors:** Yongkun Wang, Yang Zhang, Zhen Wu, Hailin Sun, Wei Zhang, Ailan Cai, Zhaoqing Cui, Shanping Sun

**Affiliations:** 1 Department of Hernia Surgery, Liaocheng People’s Hospital, Affiliated to Shandong First Medical University, Liaocheng, 252000 Shandong China; 2 Department of Anesthesiology, Liaocheng People’s Hospital, Affiliated to Shandong First Medical University, Liaocheng, 252000 Shandong China

**Keywords:** Inguinal hernia, Day surgery, Ultrasonic guidance, Local nerve block, Tension-free repair

## Abstract

The clinical characteristics of open hernia repair under local nerve block guided by ultrasound and epidural anesthesia under daytime surgery mode were compared and analyzed, and the safety, rationality and effectiveness of tension-free repair of inguinal hernia in elderly patients under local nerve block guided by ultrasound were discussed. The clinical data of 200 patients who underwent inguinal hernia day surgery in Liaocheng People's Hospital Affiliated to Shandong First Medical University from January 2022 to October 2022 were retrospectively analyzed, including 150 patients who underwent local anesthesia block surgery and 50 patients who underwent epidural surgery. The visual analog score of the ultrasound local anesthesia group was lower than that of the epidural surgery group at 4 h after operation. The time of getting out of bed and postoperative exhaust were shorter than those of epidural operation group. The recovery rate of unrestricted activity 2 weeks after surgery was higher than that in epidural surgery group (*P* < 0.05). The incidence of postoperative acute urinary retention between the two groups was lower in local ultrasound anesthesia group, and the difference was statistically significant (*P* < 0.05). The median follow-up time was 4(1–6) months, and the follow-up rate was 100%. Postoperative complications were seroma, wound infection, chronic pain and recurrence, and there was no statistical significance between the two groups (*P* > 0.05). No serious complications occurred in both groups. Compared with open epidural surgery, ultrasound-guided local nerve block tension-free day surgery in the elderly has the advantages of less pain, faster recovery, and is safe and feasible.

## Introduction

With the introduction of the concept of day surgery into the field of hernia and abdominal wall surgery, inguinal hernia has also become an important disease of day surgery. How to safely complete the day surgical treatment of inguinal hernia has become the focus of attention in the field of hernia surgery [1]. Inguinal hernia is a common disease, and elderly patients account for the highest proportion among adult inguinal hernia patients. According to a survey data based on 58 hospitals, elderly patients aged over 60 years old account for 60.8% of all hospitalized adult inguinal hernia patients undergoing surgical treatment [2]. Because daytime inguinal hernia surgery needs to be completed within 24 h and the patient can safely leave the hospital, therefore, under the daytime diagnosis and treatment mode, attention should be paid to the perioperative management of elderly inguinal hernia patients, as well as the selection and consideration of surgical and anesthetic methods, which is of great significance for improving the clinical diagnosis and treatment level of elderly inguinal hernia [3].

## Data and methods

### General data

A retrospective analysis was performed on 200 patients who underwent diurnal mode inguinal hernia repair in Liaocheng People's Hospital affiliated to Shandong First Medical University from January 2022 to December 2022. The surgical method was open Lichtenstein tension-free repair, and the patients were divided into local ultrasound anesthesia surgery group (150 cases) and epidural surgery group (50 cases). There were 189 males and 11 females. The age ranged from 60 to 89 (71.1 ± 6.3) years. According to the improved Gilbert classification of inguinal hernia (according to Guideline for diagnosis and treatment of adult groin hernia(2018 edition)) [4], there were 26 cases of type I hernia, 41 cases of type II hernia, 61 cases of type III hernia, 24 cases of type IV hernia, 39 cases of type V hernia, 7 cases of type VI hernia and 2 cases of type VII hernia. There were 124 cases complicated with cardiovascular disease, 28 cases complicated with diabetes mellitus, 53 cases complicated with prostatic hyperplasia, 42 cases complicated with respiratory system disease, 17 cases complicated with abdominal operation history, and 24 cases complicated with cerebrovascular disease. There was no statistical significance in the comparison of general information and complications between the two groups (*P *> 0.05). See Table 1 for details. All patients were discharged within 24h after surgery.

**Table 1:** General information and complications of patients

	Age(year)	Sex(male/female)	Hernia typing						
			Type I	Type II	Type III	Type IV	Type V	Type VI	Type VII
Epidural surgery group	69.82	47/3	7(14%)	10(20%)	15(30%)	5(10%)	9(18%)	2(4%)	1(2%)
Local ultrasound anesthesia surgery group	71.11	142/8	19(12.7%)	31(20.7%)	46(30.7%)	19(12.7%)	30(20%)	5(3.3%)	1(0.7%)
*P*	> 0.05	> 0.05	> 0.05	> 0.05	> 0.05	> 0.05	> 0.05	> 0.05	> 0.05

	Complication					
	Cardiovascular disease	Cerebrovascular disease	Diabetes	Respiratory system disease	Abdominal operation history	Prostatic hyperplasia
Epidural surgery group	29(58%)	5(10%)	9(18%)	11(22%)	4(8%)	12(24%)
Local ultrasound anesthesia surgery group	95(63.3%)	19(12.7%)	19(12.7%)	31(20.7%)	13(8.7%)	41(27.3%)
*P*	> 0.05	> 0.05	> 0.05	> 0.05	> 0.05	> 0.05

### Inclusion and exclusion criteria for day surgery

(1) 60 ≤ age ≤ 89 years old. (2) American Society of Anesthesiologists (ASA) classification: I–II patients with no obvious heart or lung disease, or III patients with coexisting disease stable for more than 3 months under good control. (3) No coagulation disorder. (4) Exclusion of Alzheimer's disease, mental illness and other unable to cooperate. (5) Exclude those who live alone.

### Condition assessment of day surgery

(1) The clinic shall complete the necessary routine examination and basic disease assessment. Admission, preoperative education and preoperative preparation were performed on the day of operation. (2) Preoperative anesthesia evaluation. The surgical anesthesia was epidural and local anesthesia, and the operation was carried out in accordance with the Lichtenstein surgical standard [5]. (5) After the operation, patients should be sent to the day ward after reaching the standard of anesthesia and resuscitation, where vital signs were observed and postoperative pain management was performed [6]. Antibiotics and indwelling urinary catheter were not routinely used before and after surgery [7]. (4) Outpatient follow-up was conducted 7 days, 1 month, and 3 months after surgery according to the standard of day surgery [8].

### Anesthesia and surgical methods

#### Methods of anesthesia

Epidural group: The puncture site of anesthesia was T12 – L1 or L1 – L2, and lidocaine + ropivacaine was used as anesthetic. The spinal canal puncture was successful in the epidural group.

**Fig. 1:**
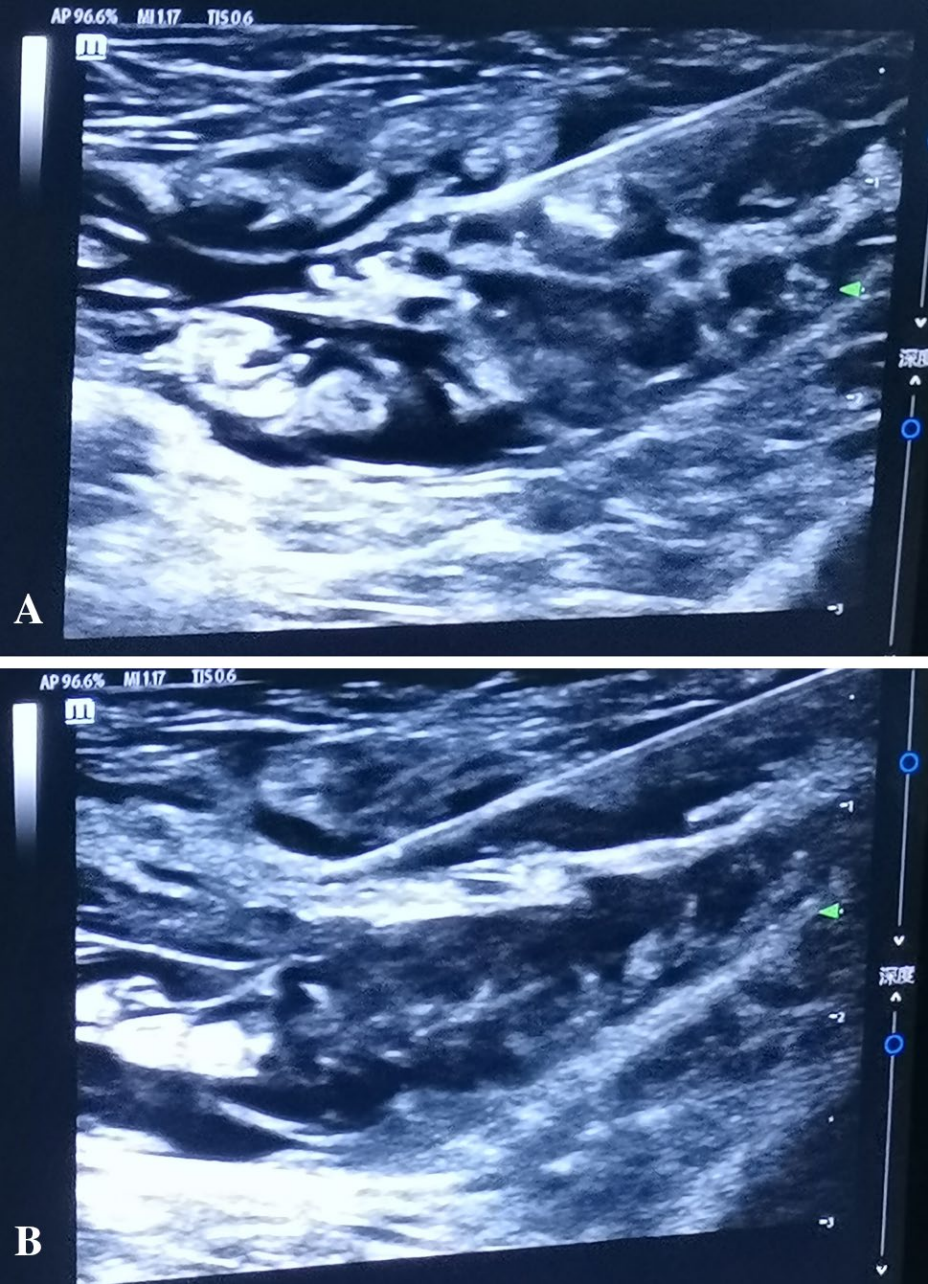
Diagram of ultrasound-guided local nerve block anesthesia. **A** The puncture needle was inserted between the internal oblique muscle and the transverse abdominal muscle. **B** The puncture needle was inserted between the external abdominal oblique and the internal abdominal oblique

Ultrasound-guided group: All procedures were performed or supervised by senior anesthesiologists skilled in ultrasound-guided puncture techniques. All patients received 2% lidocaine 30 ml and 1% ropivacaine 10 ml as local anesthetics for nerve block [9]. Linear array probes with frequencies of 6 –13 MHz are used under ultrasonic guidance. The probe was placed on the line between the anterior superior iliac spine and the navel on the affected side, and the sonographic images of the transverse abdominal muscle and the internal oblique muscle were clearly displayed. The needle was inserted about 1 cm inside the anterior superior iliac spine, and the direction and depth of the puncture needle were observed in real time using the in-plane technology, so that the puncture needle was inserted between the internal oblique muscle and the transverse abdominal muscle (Fig. 1A). When no blood was drawn, a small amount of 15–20 ml of local anesthetic was gradually injected into the nerve fascia layer to produce abdominal wall block (Fig. 1B). The operation was performed 15 min after the block was completed. Blood pressure, oxygen saturation and heart rate of all patients were monitored intraoperatively, and local infiltration of lidocaine could be assisted as required. Contraindications of anesthesia: (1) Infection, tumor and severe deformity at the puncture site. (2) Allergic patients to local anesthesia drugs. (3) Patients with severe coagulation dysfunction. (4) Peripheral neuropathy. If there are contraindications of anesthesia, choose other anesthesia methods in time.

### Statistical processing

SPSS 21.0 statistical software was used. Pearson Chi-square test and corrected Chi-square test were used for comparison of positive rates between groups. Measurement data were expressed in the form of mean ± standard deviation and *T* test was used. *P* < 0.05 was considered statistically significant.

## Results

There were a total of 200 patients in the two groups. The postoperative evaluation indexes of the two groups were shown in Table 2. The visual analog scale (VAS) at 4 h after surgery in the local anesthesia group was lower than that in the epidural surgery group (2.71 vs. 5.23, *P* < 0.05), and the time to get out of bed after surgery was shorter than that in the epidural surgery group (3.35 vs. 7.35, *P* < 0.05). The postoperative exhaust time was shorter than that of epidural surgery group (5.52 vs 10.35, *P* < 0.05), the incidence of postoperative acute urinary retention was lower than that of epidural surgery group (1.3% vs 4%, *P* < 0.05), and the recovery rate of unrestricted activity 2 weeks after surgery was higher than that of epidural surgery group (98.7% vs 94%, *P* < 0.05). The follow-up time was 1–6 months, and the follow-up rate was 100%. A total of 17 cases of postoperative seroma occurred in the two groups, all of which dissipated after intermittent aspiration or physiotherapy. One case of wound infection was cured after dressing change. After operation, 11 cases of chronic groin pain were relieved by local physiotherapy. Postoperative recurrence in 0 cases. There were no significant differences in postoperative seroma, wound infection, chronic pain and recurrence rates between the two groups (all *P* < 0.05), as shown in Table 3. During the follow-up period, there were no intestinal injury, intestinal obstruction and other serious complications in both groups.

**Table 2:** Postoperative evaluation indexes of local anesthesia operation group and epidural operation group

	Visual analog scale	Time to get out of bed (h)	Exhaust time (h)	Acute urinary retention [case(%)]	Recovery rate of unrestricted activity 2 weeks after surgery [case(%)]
Epidural surgery group	5.23 ± 1	7.35 ± 1	10.35 ± 1	2 (4%)	47(94%)
Local ultrasound anesthesia surgery group	2.71 ± 1	3.35 ± 1	5.52 ± 1	2(1.3%)	148(98.7%)
*P*	< 0.05	< 0.05	< 0.05	< 0.05	> 0.05

**Table 3:** Complications of local anesthesia group and epidural surgery group (case(%))

	Seroma	Wound infection	Chronic pain	Recurrence
Eepidural surgery group	5 (10%)	1 (2%)	3 (6%)	0
Local ultrasound anesthesia surgery group	12 (8%)	1 (0.7%)	8 (5.3%)	0
*P*	> 0.05	> 0.05	> 0.05	> 0.05

## Discussion

Daytime surgery model has developed rapidly in recent years. Day surgery mode refers to a surgical mode in which patients have completed preoperative examination before admission, make an appointment for surgery, stay in hospital on the same day, operate on the same day, and leave hospital under short-term observation. Inguinal hernia is a common clinical disease. According to statistics, every year there are more than 20 million new cases all over the world, and more than 3 million new cases every year in our country. The inguinal hernia operation plays an important role in the daytime surgery. The tension-free, minimally invasive, postoperative pain, comfort and cost of inguinal hernia treatment have always been the focus of surgeons [9]. For elderly patients, reducing anesthesia complications, reducing postoperative pain, shortening postoperative braking time and hospital stay, and resuming daily life as soon as possible are also a manifestation of minimally invasive effects. We believe that the concept of minimally invasive should not be limited to surgical methods, and it is a surgical concept and technical system to minimize the local trauma and systemic reaction caused by surgery while removing lesions as accurately as possible. Local nerve block anesthesia for elderly inguinal hernia has been recognized by physicians [10]. It can be performed for patients with heart, lung, brain and other diseases, even oral anticoagulants [11]. It not only expands surgical indications, improves surgical safety but also avoids respiratory and circulatory complications that may be caused by general anesthesia and epidural space block anesthesia. Reduce the incidence of postoperative urinary retention, compression injury caused by prolonged bed rest and precipitating pneumonia and other complications [12]. There are more and more reports on the treatment of senile inguinal hernia with local nerve block guided by ultrasound [13].

Ilioinguinal nerve and ilioinguinal inferior ventral nerve are two nerves closely related to inguinal hernia surgery, both of which send out muscular branches to jointly innervate abdominal wall muscles, while the ilioinguinal inferior ventral nerve provides cutaneous branches and terminal branches of ilioinguinal nerve to innervate sensory sensations in the inguinal region. This is the anatomical basis of local nerve block in inguinal hernia surgery. In clinical practice, it was found that the ilioinguinal nerve and ilioinferior abdominal nerve in most patients showed two oval structures between the internal oblique muscle and the transverse abdominal muscle in ultrasonic images, with low echo shadow inside and high echo shadow around the outside. Under the guidance of ultrasound, the gap between the internal oblique muscle and the transverse abdominal muscle was accurately found, and the ilioinguinal nerve and ilioinferior abdominal nerve were accurately blocked [14]. For a small number of patients with delicate nerve anatomy, variation in course and unclear nerve structure in ultrasound image, appropriate increase of local anesthetic dosage can also achieve good blocking effect by forming "water sac" between the internal oblique muscle and the transverse abdominal muscle.

This study suggests that the application of local anesthesia in tension-free inguinal hernia repair in the elderly has the following advantages: (1) Local anesthesia has little impact on human respiration and circulation, and high safety, avoiding the risks brought by epidural anesthesia catheter, and epidural anesthesia on sympathetic ganglion and spinal nerve block after vasodilation, peripheral resistance decreased, reduced cardiac blood volume, blood pressure often fluctuate significantly, so local anesthesia is particularly suitable for the elderly with cardiovascular and cerebrovascular diseases. (2) Partial anesthesia does not affect patients’ activities, avoiding bed immobility accompanied by general anesthesia or regional anesthesia, which is beneficial for preventing lung complications and lower extremity deep vein thrombosis in the elderly. (3) Partial anesthesia does not affect the bladder urine control function of patients, preoperative often do not need catheterization, postoperative urinary retention occurs less, the elderly with mild or severe prostate hyperplasia, no catheterization will not increase the pain. (4) If there is no pain after the operation, the time to get out of bed can significantly shorten the length of hospital stay and reduce the cost of hospitalization.

This study is a single-center retrospective study with limitations. More relevant data, such as case numbers, multicenter or prospective studies, are expected to support this. Nerve block anesthesia requires high technical and experience requirements of anesthesiologists and requires special training. The median follow-up time in this study was only 4 months, and longer follow-up time was needed to provide more accurate data for immediate and long-term postoperative complications.

In conclusion, compared with open surgery under epidural anesthesia, tension-free repair of senile inguinal hernia under ultrasound-guided local nerve block under daytime diagnosis and treatment mode has the advantages of less pain and faster recovery, which is safe and feasible.

## Data Availability

We confirm that we will share the data underlying the findings reported in this manuscripts and allow researchers to verify the results presented, replicate the analysis, and conduct secondary analyses.
